# Statin Use and Outcomes of Patients With Acute Ischemic Stroke Treated With Intravenous Thrombolysis: A Systematic Review and Meta-Analysis

**DOI:** 10.3389/fneur.2021.734927

**Published:** 2021-09-22

**Authors:** Yu Guo, Xinmei Guo, Kai Zhao, Qiangji Bao, Jincai Yang, Mingfei Yang

**Affiliations:** ^1^Graduate School, Qinghai University, Xining, China; ^2^Biomedical Engineering Research Center, Kunming Medical University, Kunming, China; ^3^Department of Neurosurgery, Qinghai Provincial People's Hospital, Xining, China

**Keywords:** stroke, thrombolysis, statin, intracranial hemorrhage, functional outcomes, mortality, meta-analysis

## Abstract

**Background:** The data on the relationship between statin use and clinical outcomes after intravenous thrombolysis (IVT) for acute ischemic stroke (AIS) are in controversy.

**Objective:** This systematic review and meta-analysis aimed to evaluate the safety and efficacy of statins administered prior to onset and during hospitalization in patients with AIS treated with IVT.

**Methods:** We searched PubMed, EMBASE, and the Cochrane Central Register of Controlled Trials from inception until June 8, 2021. Comparative studies investigating statin effect on intracranial hemorrhage (ICH), functional outcomes, and mortality in adults with AIS treated with IVT were screened. Random-effect meta-analyses of odds ratios (ORs) with corresponding 95% confidence intervals (CIs) were performed. The protocol was registered in PROSPERO (CRD42021254919).

**Results:** Twenty-two observational studies were included, which involved 17,554 patients. The pooled estimates showed that pre-stroke statin use was associated with a higher likelihood of symptomatic ICH (OR 1.31; 95% CI 1.07–1.59; *p* = 0.008) and any ICH (OR 1.21; 95% CI 1.03–1.43; *p* = 0.02). However, the pre-stroke statin use was not significantly associated with the 3-month mortality, 3-month favorable functional outcome (FFO, modified Rankin Scale [mRS] score 0–1), and 3-month functional independence (FI; mRS score 0–2). However, in-hospital statin use was associated with a reduced risk of symptomatic ICH (OR 0.46; 95% CI 0.21–1.00; *p* = 0.045), any ICH (OR 0.51; 95% CI 0.27–0.98; *p* = 0.04), and 3-month mortality (OR 0.42; 95% CI 0.29–0.62; *p* < 0.001) and an increased probability of 3-month FFO (OR 1.33; 95% CI 1.02–1.744; *p* = 0.04) and 3-month FI (OR 1.41; 95% C, 1.11–1.80; *p* = 0.005).

**Conclusions:** The present systematic review and meta-analysis suggests that in-hospital statin use after IVT may be safe and may have a favorable impact on clinical outcomes, a finding not observed in studies restricted to patients with pre-stroke statin use.

## Highlights

- Twenty-two observational articles with more than 15,000 patients were enrolled.
- Pre-stroke statin use probably increase the risk of intracranial hemorrhage, but has no effect on functional outcome or mortality.
- In-hospital statin use probably decrease the risk of intracranial hemorrhage and mortality and increase the odds of a good functional outcome.

## Introduction

Stroke is a common devastating neurological condition and one of the top causes of disability and mortality worldwide (1, 2). There are two major types: ischemic stroke and hemorrhagic stroke. Of note, acute ischemic stroke (AIS) accounts for ~80% of total strokes (3). In terms of treatment strategy of AIS, timely reperfusion of ischemic tissue to save the ischemic penumbra is the key to avoid severe disability and premature death (4). Intravenous thrombolysis (IVT) with recombinant tissue plasminogen activator, which is the only thrombolytic drug approved by the US Food and Drug Administration for AIS (5, 6), is considered to be most effective when administered within the first few hours of stroke onset (7).

For many years, researchers and medical doctors have been looking for a combination therapy to reduce the risk of mortality and improve functional outcomes for AIS patients treated with IVT. Statins, one of the most commonly prescribed medications for treatment of dyslipidemia, have gained attention recently as promising therapeutic agents for neurological conditions (8). Studies in animal models have shown that statins have pleiotropic effects on neuronal survival, angiogenesis, neurogenesis, and brain remodeling in ischemic stroke brain injury (9–12). Thereby, statins have potential neuroprotective and neurorestorative effects for AIS. Previous meta-analyses driven mostly by observational studies showed that statin use in AIS patients may be associated with improved functional outcome and short-term survival (13, 14). Accordingly, a recent guideline from the American Heart Association/American Stroke Association (15) recommends that AIS patients qualified for statin treatment should receive statin therapy as soon as possible. However, this recommendation is mainly based on observational studies of AIS patients with heterogeneous treatments. The existing observational studies on whether the use of statin is associated with any clinical benefit in AIS patients after IVT have reported fragmentary and conflicting results. Thus, a relatively homogeneous set of participants (AIS patients receiving IVT) was enrolled in this meta-analysis.

We hypothesize that statin use is likely to be associated with improved mortality and functional outcomes in AIS patients treated with IVT. Given that there is no randomized clinical trial (RCT) to date evaluating the safety and efficacy of statin therapy in patients with AIS treated with IVT, we performed a comprehensive systematic review and meta-analysis of *post-hoc* analyses of RCTs and observational studies to investigate its comparative safety and efficacy.

## Methods

This meta-analysis was conducted strictly in accordance with the PRISMA (Preferred Reporting Items for Systematic Reviews and Meta-Analyses) guidelines (16). It was prospectively registered in the PROSPERO (International Prospective Register of Systematic Reviews) registry, with registration number of CRD42021254919. The PRISMA checklist is available in Supplementary Table 1.

### Search Strategy

One investigator (QB) performed a comprehensive literature search in multiple electronic databases (PubMed, EMBASE, and the Cochrane Central Register of Controlled Trials) until June 8, 2021, without any restrictions. MeSH (in PubMed) and Emtree (in EMBASE) terms were used, as well as text words. Search terms included those related to stroke, thrombolysis, statins, and their variants. The detailed search strategy is available in Supplementary Table 2. Two investigators (YG and JY) manually searched all the references from relevant reviews and meta-analyses for additional studies.

### Inclusion and Exclusion Criteria

Inclusion criteria included the following: (1) types of studies: *post-hoc* analyses of RCT, prospective or retrospective cohort study; (2) characteristics of participants: adult patients (≥18 years) with AIS treated with IVT (with recombinant tissue plasminogen activator); (3) types of interventions: statin therapy regardless of type and dose; and (4) types of outcome measures: at least one outcome of interest, including symptomatic intracranial hemorrhage (ICH), any ICH, 3-month mortality, 3-month favorable functional outcome (FFO), and 3-month functional independence (FI), with odds ratio (OR) or clinical data to calculate OR.

Exclusion criteria included the following: (1) abstract with insufficient data; (2) studies that included fewer than 50 patients; (3) statin use only as a covariate in the statistical model; (4) studies providing only overlapping data with previous publication.

### Study Selection

The following study selection processes were performed. Step 1: the records obtained from initial search were imported into the Zotero citation management software (www.zotero.org) and duplicates were removed. Step 2: two investigators (YG and JY) screened the titles and abstracts of remaining articles and excluded the non-relevant articles. Step 3: the full texts of the relevant articles were retrieved for further assessment of eligibility. Disagreements were resolved through group discussion with another investigator.

### Data Extraction

Two investigators (YG and XG) independently extracted data from each included study using a standardized form. The following information was extracted: (1) study characteristics: name of first author, year of publication, country of origin, type of design, and total number of patients; (2) patient characteristics: age, sex, and baseline National Institutes of Health Stroke Scale (NIHSS) score; (3) intervention characteristics: use of statins; and (4) data on outcomes of interest, etc. Disagreements were resolved through group discussion with another investigator.

### Risk of Bias Assessment

The Newcastle–Ottawa scale (NOS) (17) was used to evaluate the methodological quality of *post-hoc* analyses of RCTs and cohort studies included in this meta-analysis. The quality control and bias assessment were performed independently by two investigators (YG and XG). NOS score >7, 7 ≥ NOS score > 5, and NOS score ≤5 indicated good quality, fair quality, and poor quality, respectively. Disagreements were resolved through group discussion with another investigator.

### Statistical Analysis

We investigated the association between statin use and clinical outcomes using pooled ORs and their corresponding 95% confidence intervals (CIs). To stabilize the variance and normalize the distribution, ORs with corresponding 95% CIs were extracted from each study and transformed into log OR and standard error (18). For studies that did not report risk estimates for the comparison of user vs. non-user of statins, we calculated ORs based on the available published data (19). Meta-analyses were performed using a random-effect model accounting for clinical heterogeneity (20). The effects of pre-stroke and in-hospital statin use were considered separately. *p* < 0.05 was considered statistically significant.

Statistical heterogeneity across studies was assessed by the Cochran Q test and quantified by the *I*^2^ statistic. For the qualitative interpretation of heterogeneity, *I*^2^ > 50% was considered significant (21). Potential publication bias across studies was graphically evaluated using a funnel plot and estimated through Egger's test (with *p* < 0.1 indicating significance) (22).

Meta-analyses were performed using RevMan 5.3 software (Nordic Cochrane Centre, Cochrane Collaboration, Copenhagen, Denmark). Egger's test was conducted with Stata 15.0 software (Stata Corporation, College Station, TX, USA).

## Results

### Literature Search and Study Selection

**Figure 1:**
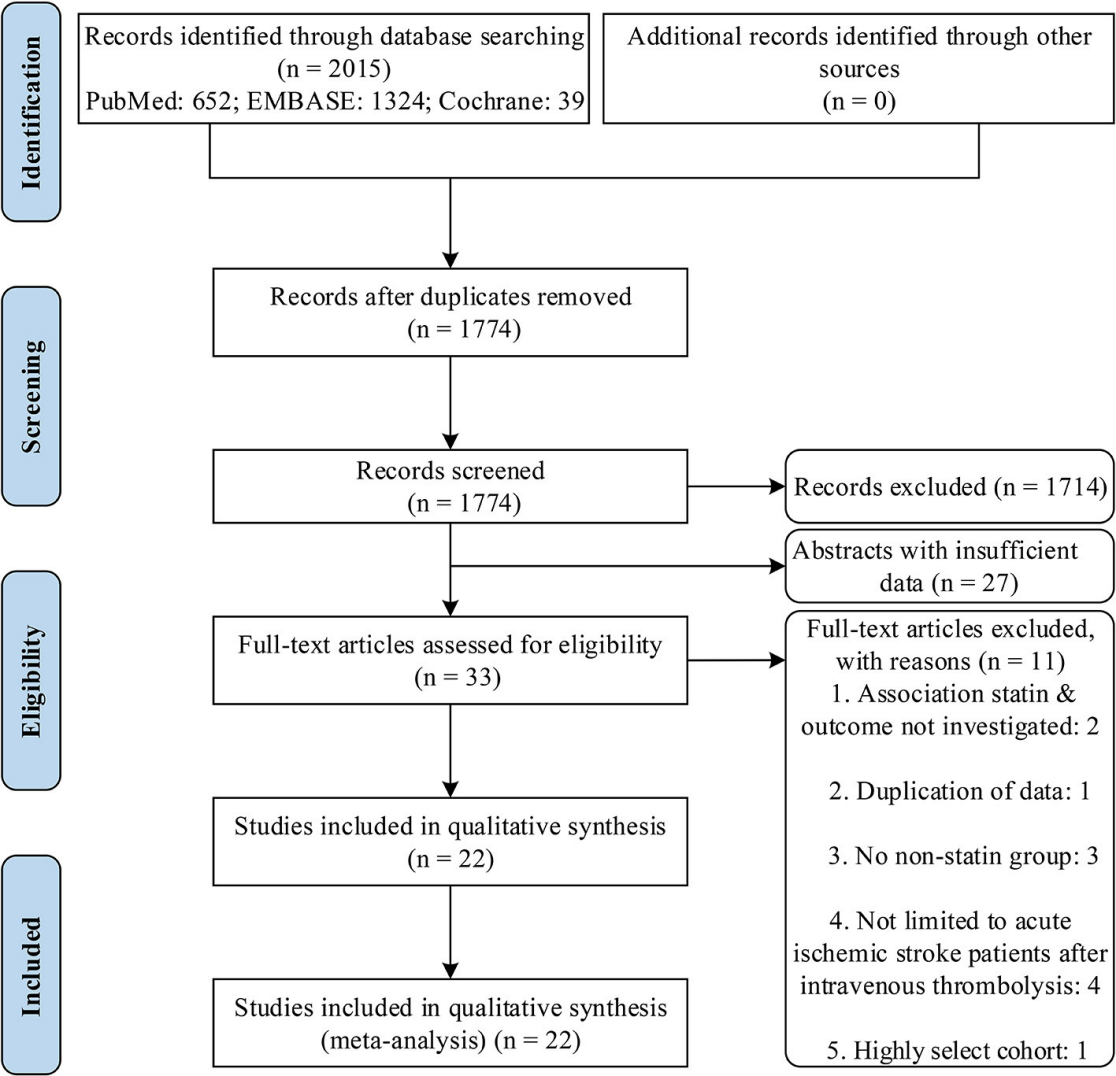
PRISMA flow diagram.

Our literature searches in the PubMed, EMBASE, and the Cochrane Central Register of Controlled Trials databases yielded 652, 1,324, and 39 records, respectively. After review of titles and abstracts, and exclusion of duplicate records, 33 potentially eligible studies were retrieved. After careful evaluation of full texts, 11 studies were excluded (data available from Supplemental Table 3), and 22 studies (23–44) were included. The study selection process is illustrated in Figure 1.

### Study Characteristics

Among the included 22 studies (23–44), there were 2 *post-hoc* analyses of RCTs (35, 40), 13 prospective cohort studies (24, 27–29, 31, 33, 34, 37–39, 42–44), and 7 retrospective cohort studies (23, 25, 26, 30, 32, 36, 40). The 22 included studies were published from 2007 to 2021, with sample sizes ranging from 55 to 4,012 participants and a total of 17,554 participants. The mean age of participants ranged from 50 to 71 years, and most of them were male. The baseline NIHSS score varied from 7 to 17. The main outcomes were ICH, functional outcomes, and mortality after at least 3 months of follow-up. Statin therapy was classified into two major types: pre-stroke statin use and in-hospital statin use. Characteristics of included studies are summarized in Table 1.

**Table 1:** Baseline characteristics of included studies.

**References**	**Country**	**Study design**	**Total-n**	**Age-y**	**Male-%**	**Baseline NIHSS**	**Exposure**	**Statin-%**	**Follow up-m**	**Outcomes**
Alvarez-Sabín et al. (23)	Spain	RC	145	72	52	17	Statin①	17.9	3	Ⓔ
Bruning et al. (24)	Germany	PC	542	72	51	11	Statin①②	26.4①, 35.7②	3	ⒶⒸ
Cappellari et al. (25)	Italy	RC	178	NR	58	NR	Statin④	35.4	3	ⒶⒺ
Cappellari et al. (26)	Italy	RC	2,072	67	58	13	Statin⑥	40.5	3	ⒶⒸⒹⒺ
Cui et al. (27)	China	PC	215	71	53	9	Statin②	83.7	3	ⒷⒺ
Engelter et al. (28)	Europe	PC	4,012	68	56	12	Statin①	22.9	3	ⒶⒷⒸⒹⒺ
Faivre et al. (29)	France	PC	101	63	59	15	Statin①	25.0	3	ⒶⒺ
Geng et al. (30)	China	RC	119	62	71	NR	Statin③	59.7	3	ⒶⒷⒸⒹ
Kang et al. (31)	Korea	PC	86	NR	NR	NR	Statin⑤	17.4	3	ⒶⒹⒺ
Makihara et al. (32)	Japan	RC	489	71	65	12	Statin①	31.7	3	ⒷⒹ
Martinez-Ramirez et al. (33)	Spain	PC	182	68	54	14	Statin①	16.3	3	ⒶⒷⒸⒺ
Miedema et al. (34)	Netherlands	PC	476	69	54	13	Statin①	20.6	3	ⒶⒺ
Montaner et al. (35)	Spain	*Post-hoc* RCT	55	NR	NR	7	Simvastatin③	49.1	3	ⒶⒷⒸⒺ
Mowla et al. (36)	USA	RC	834	71	51	12	Statin①	33.8	3	ⒶⒺ
Rocco et al. (37)	Germay	PC	1,066	73	53	12	Statin①	20.5	3	ⒶⒷⒸⒹ
Scheitz et al. (38)	Germany	PC	481	74	50	11	Statin②	17.2	3	ⒸⒺ
Scheitz et al. (39)	Germany, Switzerland	PC	1,446	75	54	11	Statin①	21.9	3	ⒶⒺ
Scheitz et al. (40)	International	*Post-hoc* RCT	2,583	68	57	14	Statin①	15.3	3	Ⓐ
Tong et al. (41)	China	RC	367	69	55	9	Statin⑥	51.2	3	ⒶⒺ
Tsivgoulis et al. (42)	International	PC	1,660	67	59	11	Statin①	22.5	3	ⒶⒸⒹⒺ
Uyttenboogaart et al. (43)	Netherlands	PC	252	68	54	12	Statin①	12.3	3	ⒶⒸⒹⒺ
Zhao et al. (44)	China	PC	193	65	64	9	Statin①	24.4	3	ⒶⒸⒹⒺ

*NR, not report; PC, prospective cohort; RC, retrospective cohort; RCT, randomized clinical trial*.

*① pre-stroke statin use; ② post-stroke statin use; ③ started statin within 12 h of stroke onset; ④ started statin within 24 h of stroke onset; ⑤ started statin within 48 h of stroke onset; ⑥ started statin within 72 h of stroke onset; Ⓐ symptomatic intracranial hemorrhage; Ⓑ any intracranial hemorrhage; Ⓒ 3 month-mortality; Ⓓ 3 month-favorable functional outcome; Ⓔ 3 month-functional independence*.

### Study Quality

Risk of bias among the *post-hoc* analyses of RCTs and cohort studies was assessed with NOS. The results showed that 15 studies were graded as good quality (25–28, 30, 32, 34–39, 41–43) and the remaining 7 studies were graded as fair quality. The overall score of the NOS was 173 of 198 (87%), which is considered to represent an overall high quality. Details of the quality assessment are shown in Table 2.

**Table 2:** Risk of bias assessment.

**References**	**Selection**				**Comparability**	**Outcome**			**Score**
	**Representativeness of the exposed cohort**	**Selection of the non-exposed cohort**	**Ascertainment of exposure**	**Demonstration that outcome of interest was not present at start of study**	Comparability of cohorts on the basis of the design or analysis ^*^	**Assessment of outcome**	**Was follow-up long enough for outcomes to occur**	**Adequacy of follow up of cohorts**	
Alvarez-Sabín et al. (23)		✩	✩	✩	✩✩	✩	✩		7
Bruning et al. (24)	✩	✩	✩	✩		✩	✩	✩	7
Cappellari et al. (25)		✩	✩	✩	✩✩	✩	✩	✩	8
Cappellari et al. (26)		✩	✩	✩	✩✩	✩	✩	✩	8
Cui et al. (27)	✩	✩	✩	✩	✩✩	✩	✩		8
Engelter et al. (28)	✩	✩	✩	✩	✩✩	✩	✩	✩	9
Faivre et al. (29)	✩	✩	✩	✩		✩	✩	✩	7
Geng et al. (30)	✩	✩	✩	✩	✩✩	✩	✩	✩	9
Kang et al. (31)		✩	✩	✩		✩	✩	✩	6
Makihara et al. (32)	✩	✩	✩	✩	✩✩	✩	✩	✩	9
Martinez-Ramirez et al. (33)	✩	✩	✩	✩		✩	✩		6
Miedema et al. (34)	✩	✩	✩	✩	✩✩	✩	✩	✩	9
Montaner et al. (35)		✩	✩	✩	✩✩	✩	✩	✩	8
Mowla et al. (36)	✩	✩	✩	✩	✩✩	✩	✩	✩	9
Rocco et al. (37)	✩	✩	✩	✩	✩✩	✩	✩	✩	9
Scheitz et al. (38)		✩	✩	✩	✩✩	✩	✩	✩	8
Scheitz et al. (39)	✩	✩	✩	✩	✩✩	✩	✩	✩	9
Scheitz et al. (40)		✩	✩	✩		✩	✩	✩	6
Tong et al. (41)		✩	✩	✩	✩✩	✩	✩	✩	8
Tsivgoulis et al. (42)	✩	✩	✩	✩	✩✩	✩	✩	✩	9
Uyttenboogaart et al. (43)	✩	✩	✩	✩	✩✩	✩	✩		8
Zhao et al. (44)		✩	✩	✩		✩	✩	✩	6
Total	13/22	22/22	22/22	22/22	32/44	22/22	22/22	18/22	173/198

* *A maximum of 2 stars can be allotted in this category; one for age, and the other for other controlled factors*.

### Association Between Statin Use and Outcomes

Table 3 provides a comprehensive overview of the association between pre-stroke or in-hospital statin use and various clinical outcomes.

**Table 3:** Overview of the safety and efficacy analyses on different endpoints.

**Outcome**	**Pre-stroke statin use**				**In-hospital statin use**			
	**Studies, *n***	**OR (95% CI)**	***p*-value**	**Heterogeneity (*I*^**2**^, *p* for Cochran Q)**	**Studies, *n***	**OR (95% CI)**	***p*-value**	**Heterogeneity (*I*^**2**^, *p* for Cochran Q)**
sICH	12	1.31 (1.07–1.59)	0.008	*I*^2^ = 20%, *p* = 0.25	5	0.46 (0.21–1.00)	0.05^*^	*I*^2^ = 0%, *p* = 0.88
Any ICH	4	1.21 (1.03–1.43)	0.02	*I*^2^ = 0%, *p* = 0.91	3	0.51 (0.27–0.98)	0.04	*I*^2^ = 0%, *p* = 0.53
Mortality (3 mo)	7	1.06 (0.74–1.51)	0.76	*I*^2^ = 64%, *p* = 0.01	5	0.42 (0.29–0.62)	<0.001	*I*^2^ = 0%, *p* = 0.44
FFO (3 mo)	6	0.93 (0.81–1.07)	0.33	*I*^2^ = 0%, *p* = 0.67	3	1.33 (1.02–1.74)	0.04	*I*^2^ = 0%, *p* = 0.72
FI (3 mo)	10	1.14 (0.86–1.52)	0.37	*I*^2^ = 66%, *p* = 0.002	7	1.41 (1.11–1.80)	0.005	*I*^2^ = 6%, *p* = 0.38

*CI, confidence interval; FFO, favorable functional outcome; FI, functional independence; ICH, intracranial hemorrhage; OR, odds ratio; sICH, symptomatic intracranial hemorrhage*.

* *The p-value was 0.045, approximately equal to 0.05*.

### Pre-stroke Statin Use and Outcomes

**Figure 2:**
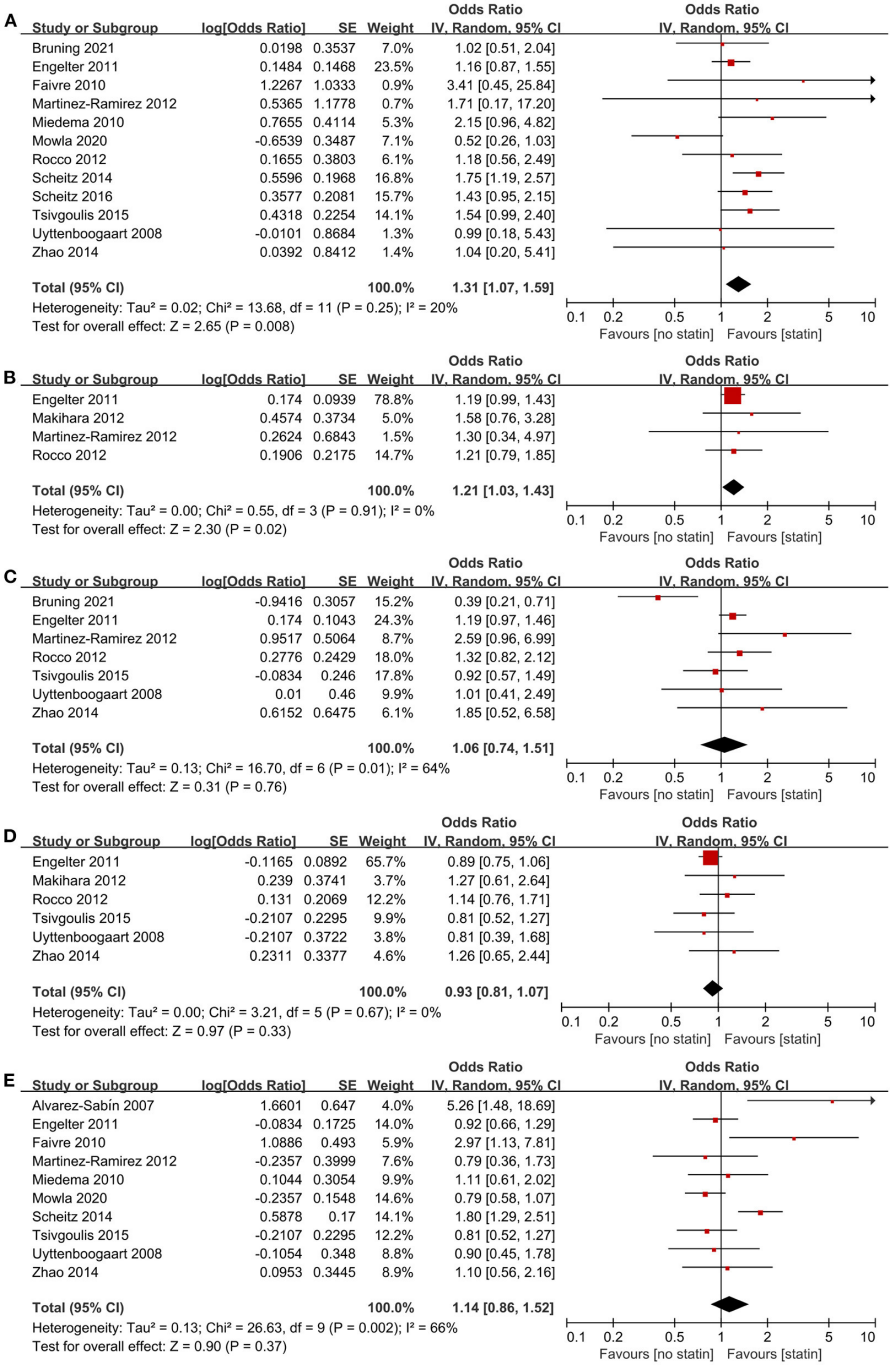
Association of pre-stroke statin use with **(A)** symptomatic intracranial hemorrhage, **(B)** any intracranial hemorrhage, **(C)** 3-month mortality, **(D)** 3-month favorable functional outcome, and **(E)** 3-month functional independence.

We identified 14 studies (23, 24, 28, 29, 32–34, 36, 37, 39, 40, 42–44) involving 13,990 participants that explored the effect of pre-stroke statin use on ICH, mortality, and functional outcome in patients with AIS treated with IVT. The pooled estimates showed that pre-stroke statin use was associated with an increased odds of symptomatic ICH (12 studies, OR 1.31; 95% CI 1.07–1.59; *p* = 0.008; *p* for Cochran Q statistic = 0.25, *I*^2^ = 20%; Figure 2A; Table 3) and any ICH (four studies, OR 1.21; 95% CI 1.03–1.43; *p* = 0.02; *p* for Cochran Q statistic = 0.91, *I*^2^ = 0%; Figure 2B; Table 3). However, pre-stroke statin use was not significantly related to 3-month mortality (seven studies, OR 1.06; 95% CI 0.74–1.51; *p* = 0.76; *p* for Cochran Q statistic = 0.01, *I*^2^ = 64%; Figure 2C; Table 3), 3-month FFO (six studies, OR 0.93; 95% CI 0.81–1.07; *p* = 0.33; *p* for Cochran Q statistic = 0.67, *I*^2^ = 0%; Figure 2D; Table 3), and 3-month FI (10 studies, OR 1.14; 95% CI 0.86–1.52; *p* = 0.37; *p* for Cochran Q statistic = 0.002, *I*^2^ = 66%; Figure 2E; Table 3).

### In-hospital Statin Use and Outcomes

**Figure 3:**
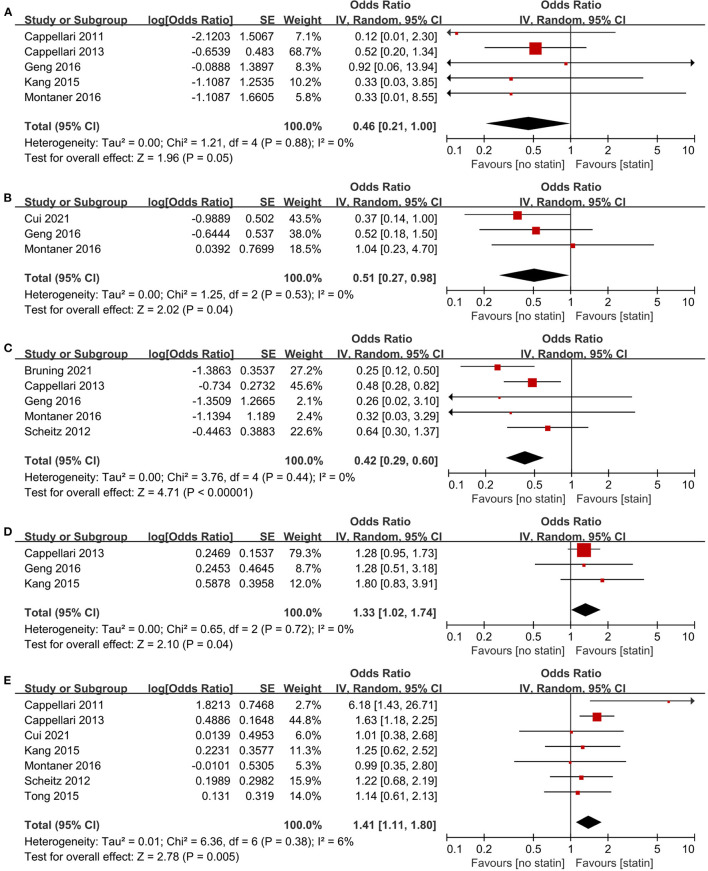
Association of in-hospital statin use with **(A)** symptomatic intracranial hemorrhage, **(B)** any intracranial hemorrhage, **(C)** 3-month mortality, **(D)** 3-month favorable functional outcome, and **(E)** 3-month functional independence.

Nine studies (24–27, 30, 31, 35, 38, 41) involving 4,115 patients reported outcomes according to in-hospital statin use. The pooled estimates showed that in-hospital statin use was associated with a lower likelihood of symptomatic ICH (five studies, OR 0.46; 95% CI 0.21–1.00; *p* = 0.045; *p* for Cochran Q statistic = 0.88, *I*^2^ = 0%; Figure 3A; Table 3), any ICH (three studies, OR 0.51; 95% CI 0.27–0.98; *p* = 0.04; *p* for Cochran Q statistic = 0.53, *I*^2^ = 0%; Figure 3B; Table 3), and 3-month mortality (five studies, OR 0.42; 95% CI 0.29–0.62; *p* < 0.001; *p* for Cochran Q statistic = 0.44, *I*^2^ = 0%; Figure 3C; Table 3). The pooled estimates also showed that in-hospital statin use was associated with 3-month FFO (three studies, OR 1.33; 95% CI 1.02–1.74; *p* = 0.04; *p* for Cochran Q statistic = 0.72, *I*^2^ = 0%; Figure 3D; Table 3) and 3-month FI (seven studies, OR 1.41; 95% CI 1.11–1.80; *p* = 0.005; *p* for Cochran Q statistic = 0.38, *I*^2^ = 6%; Figure 3E; Table 3).

### Publication Bias

For the safety and efficacy analyses on different endpoints, visual inspection of the funnel plot and the Egger statistical test revealed no evidence of asymmetry, indicating no potential publication bias (data available from Supplemental Figures 1, 2).

## Discussion

There were two major findings in this comprehensive systematic review and meta-analysis with 22 studies involving more than 15,000 participants. The primary finding was that pre-stroke statin use was associated with a potentially higher risk of systematic ICH in AIS patients treated with IVT whereas in-hospital statin use was related with a lower likelihood of symptomatic ICH. The secondary finding was that in-hospital statin use was associated with improved outcome in AIS patients treated with IVT, a finding not observed in patients using statin prior to hospital admission.

According to the American Heart Association/American Stroke Association guidelines updated in 2019 (15), it is reasonable to initiate statin therapy in eligible AIS patients. This is supported by previously published meta-analyses (13, 14), which have shown that the use of statins was associated with improved outcome. However, conflicting data were observed in a subgroup restricted to thrombolysis-treated patients (13, 14, 45). The heterogeneity in the previous studies may be due to several reasons. Firstly, a heterogeneous population undergoing different treatment modalities, including mechanical recanalization, IVT, and intra-arterial thrombolysis, was included. Secondly, the starting time of statin administration, including pre-stroke statin use and in-hospital statin use, was not considered separately. A large multicenter RCT should be the best way to address the question whether the use of statin is associated with any clinical benefit in AIS patients after IVT. Such a trial may be challenging in determining the duration and frequency of statin. However, to date, there is only one small RCT with 310 patients that has investigated the safety and efficacy of intensive statin in the acute phase of ischemic stroke after IVT therapy (46). In this trial, because of the recommendation from the American Heart Association/American Stroke Association guidelines (15), the ethics committee did not approve the no-statin group based on the principles of non-maleficence and beneficence. Therefore, we performed a comprehensive systematic review and meta-analysis of observational studies and *post-hoc* RCT analyses. Our findings may provide a good basis for determining the use of statin in combination with IVT for patients with AIS.

Our findings have important implications for both policymakers and clinicians. Firstly, previously published systematic reviews have raised concerns that statin therapy could increase the risk of ICH (47, 48). We found that in-hospital statin use probably decreased the risk of systematic ICH. Our findings provide evidence against the theoretical concerns of increased ICH risk with statin treatment. Additionally, previously published systematic reviews found that statin therapy at stroke onset was associated with improved outcome; however, inconclusive results were observed in studies restricted to thrombolysis-treated patients (13, 14, 33, 45). Our meta-analysis found that, in AIS patients receiving IVT, statin use during hospitalization was associated with improved outcome. We consider that our findings further support current international recommendations that AIS patients qualified for statin treatment should receive statin therapy as soon as possible (class of recommendation = II, level of evidence = C) (15). In addition, we believe that pretreatment with statins is not recommended as it does not improve outcomes of AIS patient treated with IVT but increases the risk of ICH.

Our findings might be attributed to the cholesterol-independent (pleiotropic) protective effects of statins. Among these, the pleiotropic effects can inhibit the differentiation of microglia to M1 cells and the release of inflammatory factors after tissue plasminogen activator treatment, thereby protecting neurovascular function. Reducing blood–brain barrier destruction may explain the positive effect of in-hospital statin treatment on the incidence of hemorrhagic transformation and clinical outcomes (49, 50). In a rat model of embolic stroke, combination treatment with atorvastatin and tissue plasminogen activator at 4 h after stroke significantly reduced the infarct volume, improved the neurologic function, and decreased the incidence of hemorrhagic transformation by decreasing neutrophil infiltration and metalloproteinase-9 expression (49). In addition, Lu et al. also found that rosuvastatin combined with tissue plasminogen activator after stroke onset prevented the activation of astrocytes and microglia and reduced the release of inflammatory factors, thereby alleviating blood–brain barrier disruption and hemorrhagic transformation severity (50). However, in stroke patients receiving IVT, the beneficial effects have not been observed consistently in prior statin users, because the beneficial effects of statins may diminish after withdrawal (51, 52), which is in agreement with one previous study (44). In addition, compared with statin treatment after thrombolysis, statin use before stroke significantly increased the fibrinolytic effect and disrupted homeostasis between coagulation and fibrinolysis (25). Hence, it might be possible that pre-stroke statin use associates with a potential higher risk of systematic ICH in AIS patients treated with IVT.

Certain limitations of the present study warrant further consideration. Firstly, this is a meta-analysis of observational studies. Our findings were exclusively based on data of observational studies that predispose to inherent biases, especially selection bias. Secondly, despite the use of adjusted ORs whenever applicable, unmeasured confounders cannot be eliminated due to a lack of individual study patient data. It is possible that differences in cardiovascular risk factors might account for observed associations, while the confounding role of pharmacologic differences in statins cannot be excluded. Thirdly, specific data for statin, including dosage, duration, compliance, pharmacokinetics, and statin type, were not assessed. These parameters could have introduced unmeasured biases in our analysis.

Our study also has several strengths. Firstly, to our knowledge, this is the first systematic review and meta-analysis to explore the effects of starting time of statin administration (pre-stroke or in-hospital) in patients with AIS treated with IVT. Secondly, the majority of the included studies were prospective cohort studies or *post-hoc* analysis of RCTs with high quality and had adequately adjusted for confounders. This might reduce the influences of other cardiovascular risk factors on the association of pre-stroke statin use with clinical outcomes. Thirdly, the number of available studies and the sample size were large, which allowed us to explore the association of pre-stroke and in-hospital statin administration with clinical outcomes.

## Conclusion

In AIS patients treated with IVT, pre-stroke statin use was probably associated with increased risk of ICH, but had no effect on good functional outcome or mortality at 3 months. On the contrary, in-hospital statin use probably decreased the risk of ICH and 3-month mortality and was associated with good functional outcome at 3 months.

## Data Availability Statement

The original contributions presented in the study are included in the article/Supplementary Material, further inquiries can be directed to the corresponding author.

## Author Contributions

YG: study concept and design, acquisition of data, analysis and interpretation, and critical revision of the manuscript for important intellectual content. XG: acquisition of data, analysis and interpretation, and critical revision of the manuscript for important intellectual content. KZ: critical revision of the manuscript for important intellectual content. QB and JY: acquisition of data. MY: study supervision and critical revision of the manuscript for important intellectual content. All authors contributed to the article and approved the submitted version.

## Funding

This research was funded by the Science and Technology Department of Qinghai Province (Grant No. 2019-ZJ-7040) and the National Key R&D Program of China (Grant Nos. 2018YFC1312600 and 2018YFC1312601).

## Conflict of Interest

The authors declare that the research was conducted in the absence of any commercial or financial relationships that could be construed as a potential conflict of interest.

## Publisher's Note

All claims expressed in this article are solely those of the authors and do not necessarily represent those of their affiliated organizations, or those of the publisher, the editors and the reviewers. Any product that may be evaluated in this article, or claim that may be made by its manufacturer, is not guaranteed or endorsed by the publisher.

## Abbreviations

AIS: acute ischemic stroke
CI: confidence interval
FFO: favorable functional outcome
FI: functional independence
ICH: intracranial hemorrhage
IVT: intravenous thrombolysis
mRS: modified Rankin Scale
NIHSS: National Institutes of Health Stroke Scale
NOS: Newcastle-Ottawa scale
OR: odds ratio
PRISMA: Preferred Reporting Items for Systematic Reviews and Meta-Analyses
PROSPERO: International Prospective Register of Systematic Reviews
RCT: randomized clinical trial.
